# Regulation of hepatic pyruvate dehydrogenase phosphorylation in offspring glucose intolerance induced by intrauterine hyperglycemia

**DOI:** 10.18632/oncotarget.14837

**Published:** 2017-01-27

**Authors:** Yong Zhang, Ying Zhang, Guo-Lian Ding, Xin-Mei Liu, Jianping Ye, Jian-Zhong Sheng, Jianxia Fan, He-Feng Huang

**Affiliations:** ^1^ International Peace Maternity and Child Health Hospital, School of Medicine, Shanghai Jiao Tong University, Shanghai 200030, China; ^2^ Institute of Embryo-Fetal Original Adult Disease, School of Medicine, Shanghai Jiao Tong University, Shanghai 200030, China; ^3^ Antioxidant and Gene Regulation Laboratory, Pennington Biomedical Research Center, Louisiana State University System, Baton Rouge, LA 70808, USA; ^4^ Department of Pathophysiology, School of Medicine, Zhejiang University, Hangzhou 310058, China

**Keywords:** gestational diabetes mellitus, offspring, pyruvate dehydrogenase, glucose intolerance, phosphorylation

## Abstract

**Aim:**

Gestational diabetes mellitus (GDM) has been shown to be associated with a high risk of diabetes in offspring. In mitochondria, the inhibition of pyruvate dehydrogenase (PDH) activity by PDH phosphorylation is involved in the development of diabetes. We aimed to determine the role of PDH phosphorylation in the liver in GDM-induced offspring glucose intolerance.

**Results:**

PDH phosphorylation was increased in lymphocytes from the umbilical cord blood of the GDM patients and in high glucose-treated hepatic cells. Both the male and female offspring from GDM mice had elevated liver weights and glucose intolerance. Further, PDH phosphorylation was increased in the livers of both the male and female offspring from GDM mice, and elevated acetylation may have contributed to this increased phosphorylation.

**Materials and methods:**

We obtained lymphocytes from umbilical cord blood collected from both normal and GDM pregnant women. In addition, we obtained the offspring of streptozotocin-induced GDM female pregnant mice. The glucose tolerance test was performed to assess glucose tolerance in the offspring. Further, Western blotting was conducted to detect changes in protein levels.

**Conclusions:**

Intrauterine hyperglycemia induced offspring glucose intolerance by inhibiting PDH activity, along with increased PDH phosphorylation in the liver, and this effect might be mediated by enhanced mitochondrial protein acetylation.

## INTRODUCTION

Increasing research suggests that exposure to an abnormal uterine environment during fetal development can lead to chronic health problems later in life, such as diabetes and hypertension [1]. Intrauterine hyperglycemia is a major characteristic of gestational diabetes mellitus (GDM), which has been suggested to be a vital determining factor for the risk of diabetes during adulthood for offspring [2, 3]. Our previous studies have demonstrated that metabolic imprinting resulting from a diabetic intrauterine environment, not only in the maternal line but also in the paternal line, can affect the health of offspring [4, 5]. Thus, it is of vital importance to explore the mechanisms involved in the association between intrauterine hyperglycemia and a high risk of diabetes in offspring.

Glucose intolerance is the primary cause of type 2 diabetes, and it occurs several years before the development of this disease in humans. Several factors have been proposed to explain the mechanisms of glucose intolerance, including mitochondrial dysfunction, inflammation, genetic background, oxidative stress, endoplasmic reticulum (ER) stress, lipotoxicity/hyperlipidemia, fatty liver, hypoxia, and lipodystrophy [6]. The mitochondrion is the primary subcellular organ for the oxidation and metabolism of fatty acids and glucose, and a reduction in mitochondrial function may contribute to lipid accumulation and promote glucose intolerance [7]. Mitochondrial function is decreased in patients with type 2 diabetes [8]. Furthermore, reductions in mitochondrial content result in decreased mitochondrial function in insulin-resistant offspring of type 2 diabetic parents [9]. Thus, we hypothesized that intrauterine hyperglycemia may affect the functions of key mitochondrial enzymes in offspring, thereby inducing glucose intolerance.

Pyruvate dehydrogenase (PDH) is a mitochondrial protein complex containing three types of enzymes: pyruvate dehydrogenase E1 (PDHe1), dihydrolipoamide transacetylase E2 (PDHe2) and dihydrolipoamide dehydrogenase E3 (PDHe3) [10]. PDH activity stimulates acetyl-CoA production from pyruvate in the glucose catabolism pathway, and this activity is required for insulin-induced glucose utilization. Inhibition of PDH activity by phosphorylation of the PDHe1 alpha subunit is a major mechanism of glucose intolerance in starvation and diabetes [11–13]. Induction of the phosphorylation and acetylation of mitochondrial proteins may contribute to the negative regulation of mitochondrial function [14]. However, the specific changes in PDH phosphorylation in liver tissues of offspring from GDM mothers (F1-GDM) remain unknown and need to be determined.

In the present study, we hypothesized that intrauterine hyperglycemia may increase PDH phosphorylation in offspring livers, resulting in the induction of glucose intolerance. We found that PDH phosphorylation was elevated in the lymphocytes of umbilical cord blood from GDM patients compared with those from normal pregnant women. We confirmed these results in high glucose-treated HepG2 cells. We also found that intrauterine hyperglycemia caused an elevation in PDH phosphorylation in the livers of both male and female F1-GDM mice, which may have contributed to the glucose intolerance of these mice. Furthermore, we found that induction of acetylation in the livers of F1-GDM mice potentially increased PDH phosphorylation. The results of our study indicate that intrauterine hyperglycemia may increase liver PDH phosphorylation, thereby inducing glucose intolerance in offspring.

## RESULTS

### PDH phosphorylation in umbilical cord blood lymphocytes from GDM patients

**Figure 1:**
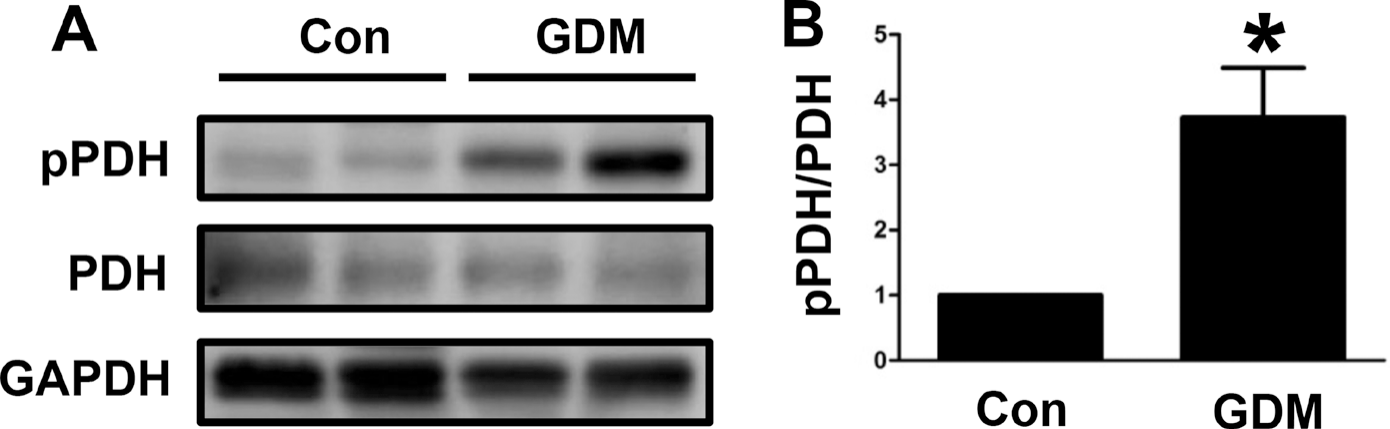
PDH phosphorylation in umbilical cord blood lymphocytes from GDM patients (**A**) Representative Western blot showing PDH phosphorylation levels in umbilical cord blood lymphocytes from control pregnant women (Con) and GDM patients. (**B**) The PDH phosphorylation levels are presented as bars. The results are presented as the mean ± SE (*n* = 4). *p < 0.05 compared with the controls.

We examined the changes in PDH phosphorylation in umbilical cord blood lymphocytes from both normal pregnant women and GDM women. We found that PDH phosphorylation was increased in the lymphocytes from the GDM women compared with those from the normal pregnant women (Figure 1A and 1B). These results indicated that the GDM patients had decreased PDH activity in the lymphocytes of umbilical cord blood that potentially impacted offspring health.

### Changes in PDH phosphorylation in high glucose-treated HepG2 cells

**Figure 2:**
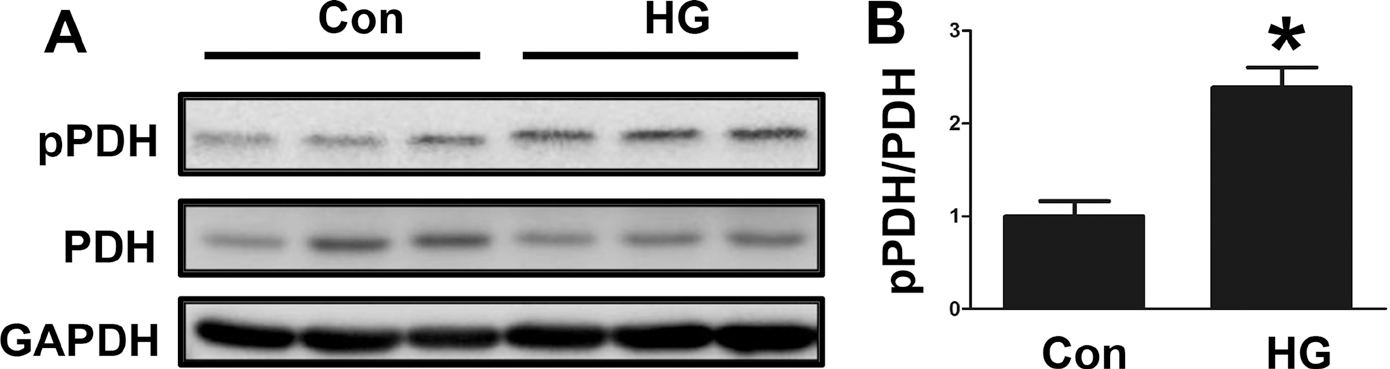
Changes in PDH phosphorylation in high glucose-treated HepG2 cells (**A**) PDH phosphorylation levels in control (Con) and high glucose (HG)-treated HepG2 cells, as determined by Western blotting. (**B**) The PDH phosphorylation signal intensities following HG treatment are presented as bars. The results are presented as the mean ± SE (*n* = 3). *p < 0.05 compared with the controls.

We further examined the changes in PDH phosphorylation by performing an *in vitro* experiment. The HepG2 cell line was treated with high glucose, and then the phosphorylated PDH level was determined by Western blotting. As shown in Figure 2A and 2B, the phosphorylated PDH level was significantly increased by the high glucose treatment. These *in vitro* results were consistent with the clinical results.

### Phenotypes of male and female F1-GDM mice

**Figure 3:**
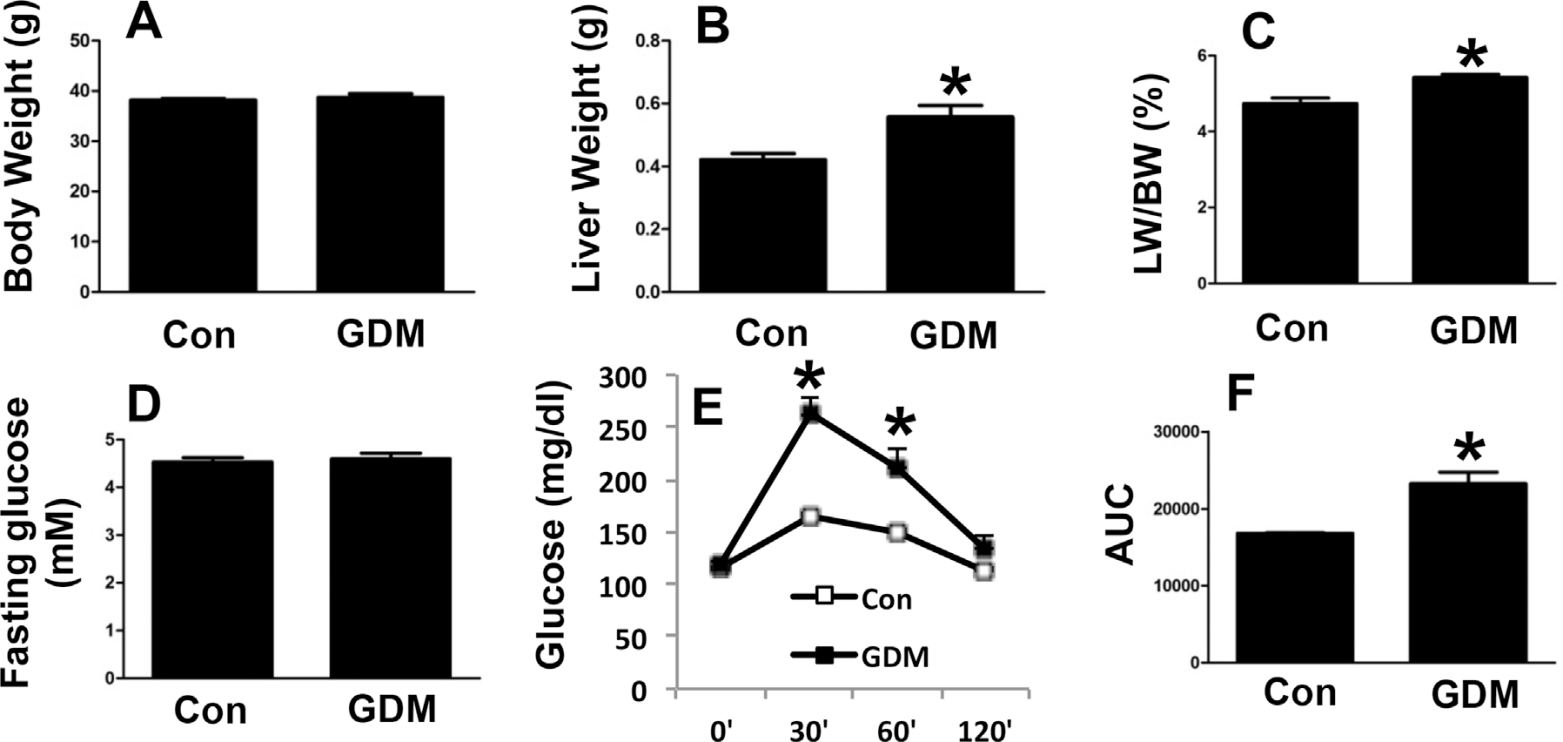
Phenotypes of male F1-GDM mice (**A**) Body weights of male control and F1-GDM mice at 8 weeks. (**B**) Liver weights of male control and F1-GDM mice. (**C**) The percentages of liver weight to body weight. (**D**) Fasting glucose levels in male control and F1-GDM mice. (**E**) Glucose tolerance test (GTT) results for male control and F1-GDM mice. (**F**) Areas under the curve (AUCs) for the GTT. In the bar graph, the results are presented as the mean ± SE (*n* = 5). *p < 0.05 compared with the control.

We next examined the phenotypes of the male F1-GDM mice and found that body weight did not markedly differ between the male F1-GDM mice and control mice (Figure 3A). Interestingly, the liver weight of the male F1-GDM mice was increased compared with that of the control mice, and the percentage of liver to body weight was also elevated (Figure 3B and 3C). The fasting glucose level did not significantly differ between the male F1-GDM mice and control mice (Figure 3D). The GTT revealed that the glucose level was dramatically increased in the F1-GDM mice compared with the control mice at 30 min and 60 min after glucose induction and that the AUC was also significantly increased (Figure 3E and 3F).

**Figure 4:**
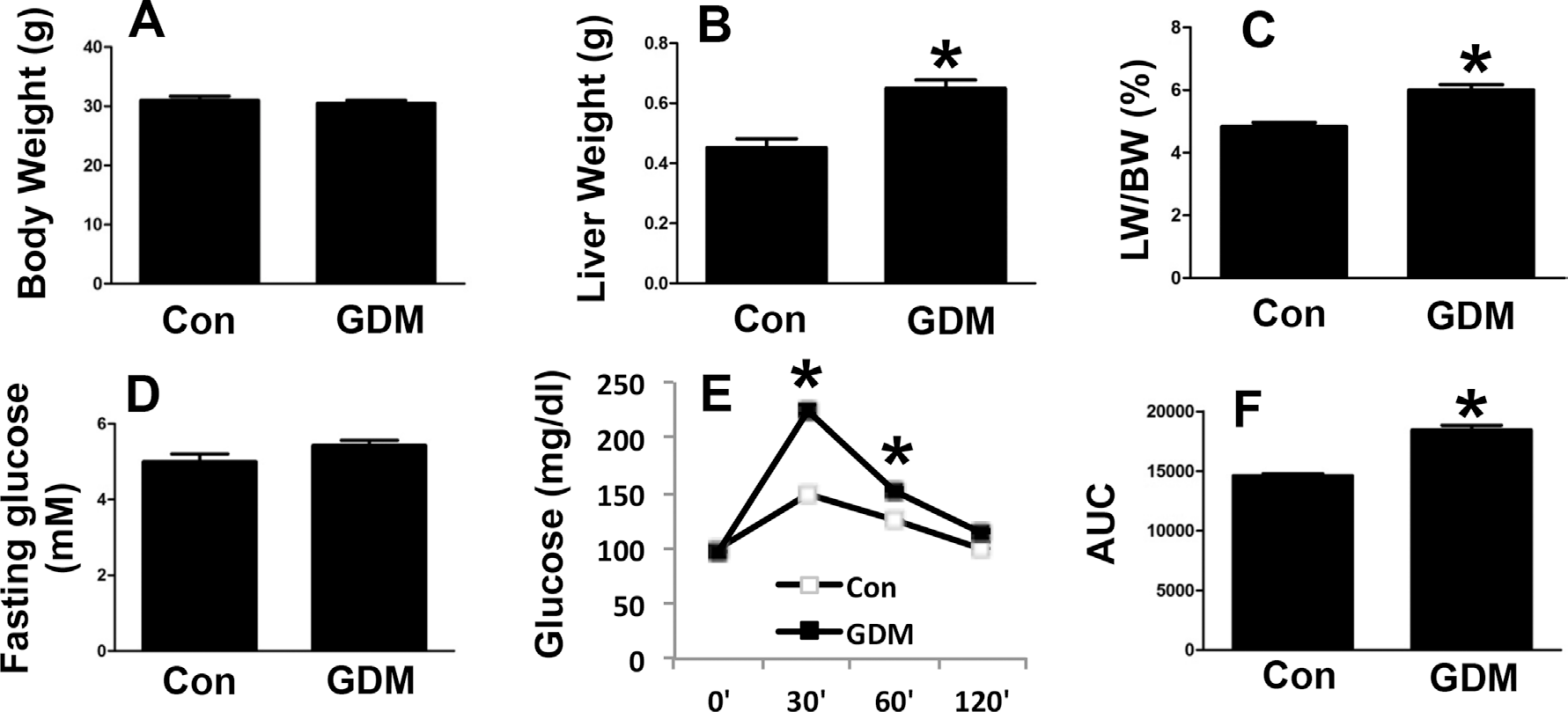
Phenotypes of female F1-GDM mice (**A**) Body weights of female control and F1-GDM mice at 8 weeks. (**B**) Liver weights of female control and F1-GDM mice. (**C**) The percentages of liver weight to body weight. (**D**) Fasting glucose levels in female control and F1-GDM mice. (**E**) Glucose tolerance test (GTT) results for female control and F1-GDM mice. (**F**) Areas under the curve (AUCs) for the GTT. In the bar graph, the results are presented as the mean ± SE (*n* = 5). *p < 0.05 compared with the controls.

Next, we examined the phenotypes of the female F1-GDM mice. Similar to the male F1-GDM mice, the female mice also had a higher liver weight and percentage of liver weight to body weight than the control mice, with no significant difference in body weight (Figure 4A–4C). In addition, no significant difference in the fasting glucose level was detected between the female F1-GDM and control mice (Figure 4D). The GTT revealed that the glucose level and AUC were elevated in the female F1-GDM mice compared with the control mice (Figure 4E and 4F). These results indicated that both the male and female F1-GDM mice had impaired hepatic function and glucose tolerance.

### Changes in PDH phosphorylation in the livers of F1-GDM mice

**Figure 5:**
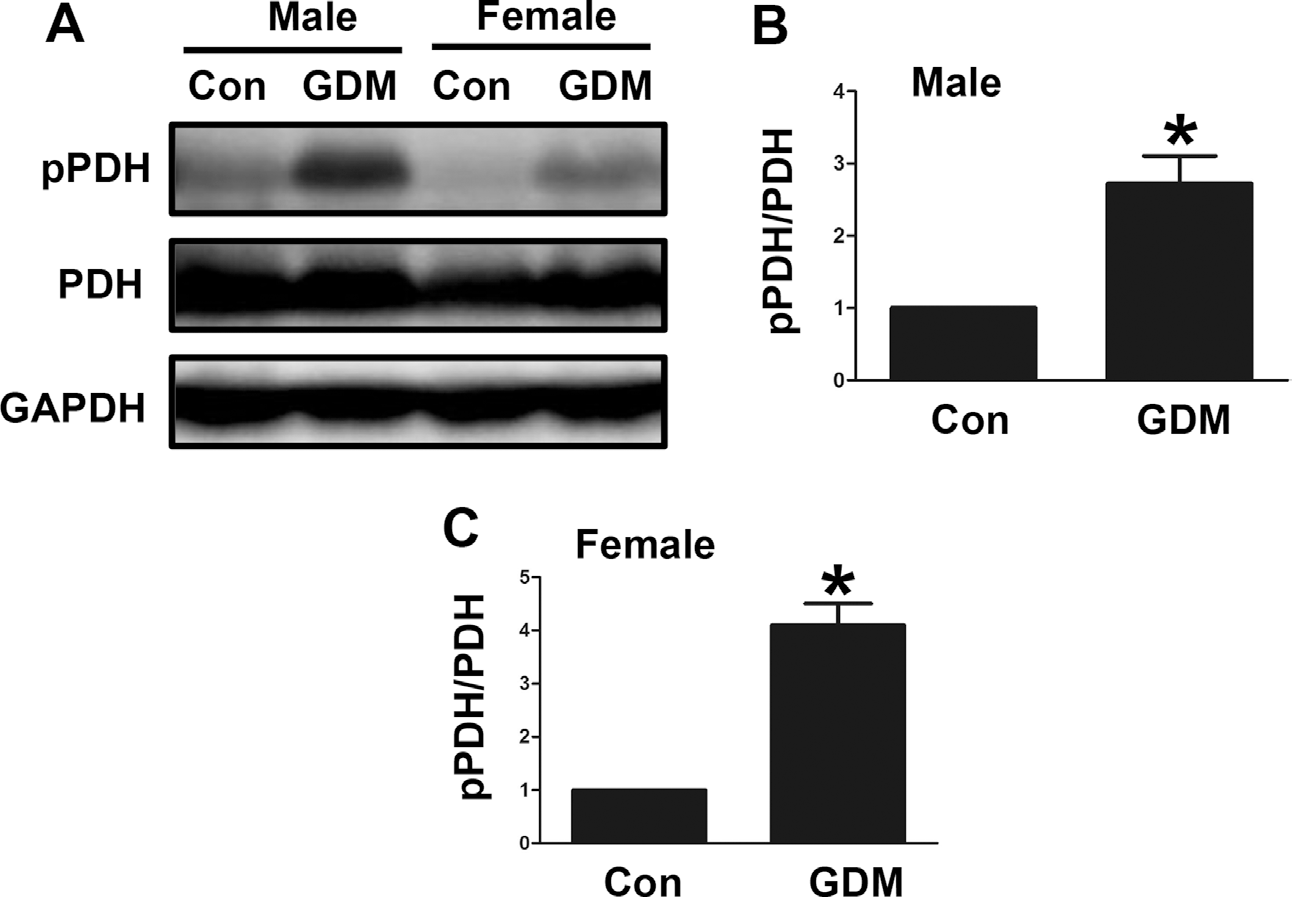
Changes in PDH phosphorylation in the livers of F1-GDM mice (**A**) PDH phosphorylation in liver tissues of both the male and female mice. (**B**) PDH phosphorylation signal intensities for the male mice are presented as bars. (**C**) PDH phosphorylation signal intensities for the female mice are presented as bars. The experiments were performed three times with consistent results, and representative blots are shown. The results are presented as the mean ± SE (*n* = 5). *p < 0.05 compared with the controls.

In the present study, we examined PDH phosphorylation in the livers of both male and female F1-GDM mice. We found that PDH phosphorylation was significantly increased in the livers of both the male and female F1-GDM mice (Figure 5A, 5B and 5C). These findings indicated that changes in PDH phosphorylation in the livers of these mice might have contributed to their impaired glucose tolerance.

### Changes in acetylation in the livers of F1-GDM mice

**Figure 6:**
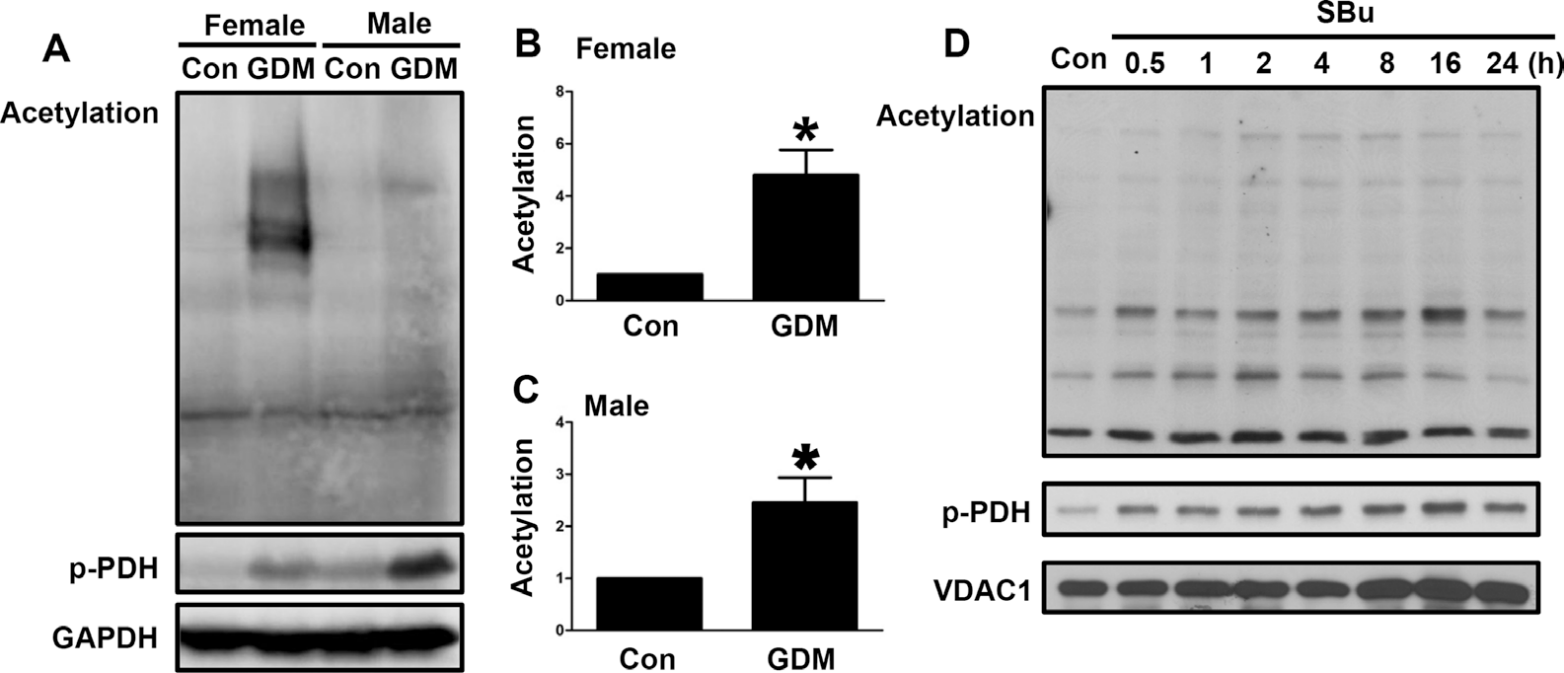
Changes in acetylation in the livers of F1-GDM mice (**A**) Acetylation and PDH phosphorylation in the livers of male and female control and F1-GDM mice, as determined by Western blotting. (**B**) Acetylation signal intensities for the female control and GDM mice are presented as bars. (**C**) Acetylation signal intensities for the male control and GDM mice are presented as bars. (**D**) Acetylation and PDH phosphorylation in liver cells treated with sodium butyrate (SBu) for different durations. The results are presented as the mean ± SE (*n* = 5). *p < 0.05 compared with the controls.

We further examined the acetylation levels in the livers of both the male and female F1-GDM mice to determine whether elevated PDH phosphorylation was associated with increased acetylation. The acetylation levels were significantly increased in the livers of the female and male F1-GDM mice compared with those of the control mice (Figure 6A, 6B and 6C). PDH phosphorylation was also enhanced in the F1-GDM mice (Figure 6A). The use of SBu, an HDAC inhibitor, to induce acetylation in mitochondria also resulted in increased PDH phosphorylation (Figure 6D). These results suggested that increased acetylation might have induced the increased PDH phosphorylation in the livers of the F1-GDM mice.

## DISCUSSION

In the present study, we have provided the first evidence that intrauterine hyperglycemia induces offspring glucose intolerance through the inhibition of PDH activity via increased PDH phosphorylation through both *in vivo* and *in vitro* experiments. Our results have revealed evidence of novel biological effects of mitochondria in glucose intolerance induced by intrauterine hyperglycemia in offspring. The results have also provided new therapeutic targets for preventing glucose intolerance in offspring of GDM mothers.

Our previous reports have demonstrated that intrauterine hyperglycemia can induce the transgenerational transmission of glucose intolerance [4]. The results of the present study have revealed that intrauterine hyperglycemia induces glucose intolerance in offspring partly through the inhibition of liver PDH activity, as PDH phosphorylation in the livers of offspring was dramatically increased by intrauterine hyperglycemia. Therefore, we speculate that both gene imprinting and posttranslational protein modifications contribute to glucose intolerance in the offspring of GDM mothers. However, the mechanism by which hyperglycemia inhibits PDH activity in the liver is not fully known and needs to be further explored in a future study.

In this study, we have shown that intrauterine hyperglycemia causes increased PDH phosphorylation in the livers of offspring. Although PDH activation has been reported to alter oxidative substrate selection to induce insulin resistance in skeletal muscles [15], it has been confirmed that PDH activity is decreased in the liver in response to insulin resistance [16]. PDH activity has been shown to be reduced in various tissues of animals and patients with diabetes or obesity-related conditions [16, 17]. PDH activity was also reduced in growth hormone-induced insulin resistance in human subjects [18]. It is possible that increased PDH phosphorylation in the liver of humans leads to decreased PDH activity and glucose intolerance. Further, PDH phosphorylation has been demonstrated to be induced by the incubation of a mitochondrial lysate with acetyl-CoA *in vitro*, with no change in the expression of PDH kinase 2 (PDK2) or 4 (PDK4) [14]. Considering the results of both this and previous studies, it is possible that hyperglycemia induces the acetylation of mitochondrial proteins, resulting in increased PDH phosphorylation, ultimately leading to glucose intolerance.

Mitochondrial dysfunction has been associated with the development of obesity and diabetes [9, 19–21]. Although the evidence of a causal relationship between mitochondrial function and diabetes remains weak, emerging evidence indicates that boosting mitochondrial function might be beneficial to patient health [22]. Mitochondrial dysfunction leads to metabolic inflexibility, with the consequence of acetyl-CoA accumulation, which inhibits PDH activity and reduces the glycolytic flux [23, 24]. GDM mothers have mitochondrial dysfunction and metabolic inflexibility, and these adverse effects can be passed on to subsequent generations. Thus, it is of vital importance to enhance mitochondrial function in GDM mothers to improve the metabolic flexibility and glucose tolerance of their offspring. The present study should aid in the exploration of novel therapies for preventing glucose intolerance in offspring affected by intrauterine hyperglycemia and thus lower the risk of diabetes development during adulthood.

## MATERIALS AND METHODS

### Cells and reagents

The HepG2 cell line was purchased from the American Type Culture Collection (Manassas, VA) and was maintained in DMEM supplemented with 10% fetal calf serum. Sodium butyrate (SBu) was purchased from Sigma (St. Louis, MO). Antibodies to phosphorylated PDH e1 alpha subunit (S293) (ab177461), PDHe1 alpha subunit (ab110330), voltage-dependent anion channel (VDAC1, ab14734) and GAPDH (ab9484) were obtained from Abcam (Cambridge, MA). In addition, an antibody to acetylated lysine (#9814) was acquired from Cell Signaling (Danvers, MA).

### GDM animal models

All GDM animal protocols were reviewed and approved by the Shanghai Jiaotong University Animal Care and Use Committee. At the age of 8 weeks, virgin female ICR mice were mated with normal males. Onset of pregnancy was determined by the presence of a copulation plug after overnight mating. After a 12-h fast, the females were randomly divided into a control group and an intrauterine hyperglycemia group with GDM (GDM group). The mice in the GDM group were administered a single intraperitoneal injection of streptozotocin (STZ; Sigma, St. Louis, MO) in 0.1 mmol/L citrate buffer (pH 4.5) at a dose of 150 mg/kg body weight. The control pregnant females received an equal volume of citrate buffer. On day 3 (D3) of pregnancy, diabetes, defined as a glucose level of between 14 and 19 mmol/L, was confirmed by measurement of the blood glucose concentration via the tail vein, and it was also measured on D7 and D20 of pregnancy to confirm the the diagnosis of diabetes as previously described [25, 26]. The pregnant mice were allowed to deliver spontaneously. The litter size was randomly reduced to 10 at birth to assure uniformity. The first generation of GDM mice (F1-GDM) were fostered by normoglycemic female mice until they were weaned at 3 weeks of age.

### *In vivo* glucose tolerance test (GTT)

Intra-peritoneal glucose injection (2 g/kg body weight) was performed in unrestrained conscious mice after a 12-h overnight fast, and the glucose level in tail vein blood was measured at 0, 30, 60 and 120 min after glucose injection using a glucometer. The area under the curve (AUC) for glucose against time was calculated for analysis of glucose tolerance, as previously described [27].

### Western blot analysis

Cell preparations were sonicated in lysis buffer, and 50 mg protein from each sample was resolved on an 8% SDS-PAGE gel and electroblotted onto a PVDF membrane. The membrane was then blocked with 5% nonfat milk containing 0.05% Tween-20, rinsed with phosphate-buffered saline (PBS; pH 7.4), and incubated with the following antibodies: rabbit polyclonal anti-phosphorylated PDH (1:10000), mouse monoclonal anti-PDH (1:1000), mouse monoclonal anti-acetylated lysine (1:1000), rabbit polyclonal anti-GAPDH (1:1000), and mouse monoclonal anti-voltage-dependent anion channel (VDAC1) (1:1000). GAPDH was probed as a loading control, while VDAC1 was probed as a mitochondrial loading control. Immunoreactivity was visualized using an enhanced chemiluminescence (ECL) substrate system. Films were scanned and were subsequently analyzed by measuring the optical densities of immunostained bands using an image processing and analysis system (Bio-Rad, Hercules, CA).

### Mitochondrial isolation

Mitochondria were isolated from fresh cells according to the standard protocol [28] using a mitochondrial isolation buffer composed of 70 mM sucrose, 210 mM mannitol, 5 mM HEPES, 1 mM EGTA and 0.5% fatty acid-free BSA (pH 7.2), and a mitochondrial assay solution (MAS) containing 70 mM sucrose, 220 mM mannitol, 10 mM KH_2_PO4, 5 mM MgCl_2_, 2 mM HEPES, 1 mM EGTA and 0.2% fatty acid-free BSA (pH 7.2, 37°C).

Fresh cells were harvested and were then homogenized using a glass-Teflon potter and centrifuged at 600 *g* for 10 min at 4°C. The resulting supernatants were centrifuged at 7,000 *g* for 10 min at 4°C, and the pellets were washed with ice-cold buffer. Following a second centrifugation at 7,000 *g* for 10 min at 4°C, the pellets containing mitochondria were suspended for further analysis. Protein concentrations were determined by the bicinchoninic acid (BCA) method, using BSA as a standard.

### Isolation of lymphocytes from umbilical cord blood

Umbilical cord blood was collected from both normal pregnant women and GDM pregnant women after delivery of their babies at the International Peace Maternity and Child Hospital, with approval of the Shanghai Jiaotong University Ethics Committee. The blood was diluted with sterile PBS and poured carefully onto a Ficoll gradient solution. The tubes were centrifuged at 400 *g* for 20 min, and the lymphocyte-containing bands were isolated without touching the Ficoll solution. The lymphocytes were then washed twice with PBS and used in further analyses.

### Statistical analysis

In this study, the data were presented as the mean ± SEM from multiple samples, and each experiment was conducted at least three times with consistent results. Two-tailed, unpaired Student's *t-test* was used for statistical analysis of the data, with a significance level of *p* < 0.05.

## Abbreviations

PDH: Pyruvate Dehydrogenase
GDM: Gestational Diabetes Mellitus
GTT: Glucose Tolerance Test
AUC: Area Under Curve
SBu: Sodium Butyrate
HDAC: Histone Deacetylase
STZ: Streptozotocin
VDAC: voltage-dependent anion channel
